# Intake of Raw Fruits and Vegetables Is Associated With Better Mental Health Than Intake of Processed Fruits and Vegetables

**DOI:** 10.3389/fpsyg.2018.00487

**Published:** 2018-04-10

**Authors:** Kate L. Brookie, Georgia I. Best, Tamlin S. Conner

**Affiliations:** Department of Psychology, University of Otago, Dunedin, New Zealand

**Keywords:** fruits, vegetables, mental health, well-being, diet, mood

## Abstract

**Background:** Higher intakes of fruits and vegetables, rich in micronutrients, have been associated with better mental health. However, cooking or processing may reduce the availability of these important micronutrients. This study investigated the differential associations between intake of raw fruits and vegetables, compared to processed (cooked or canned) fruits and vegetables, and mental health in young adults.

**Methods:** In a cross-sectional survey design, 422 young adults ages 18–25 (66.1% female) living in New Zealand and the United States completed an online survey that assessed typical consumption of raw vs. cooked/canned/processed fruits and vegetables, negative and positive mental health (depressive symptoms, anxiety, negative mood, positive mood, life satisfaction, and flourishing), and covariates (including socio-economic status, body mass index, sleep, physical activity, smoking, and alcohol use).

**Results:** Controlling for covariates, raw fruit and vegetable intake (FVI) predicted reduced depressive symptoms and higher positive mood, life satisfaction, and flourishing; processed FVI only predicted higher positive mood. The top 10 raw foods related to better mental health were carrots, bananas, apples, dark leafy greens like spinach, grapefruit, lettuce, citrus fruits, fresh berries, cucumber, and kiwifruit.

**Conclusions:** Raw FVI, but not processed FVI, significantly predicted higher mental health outcomes when controlling for the covariates. Applications include recommending the consumption of raw fruits and vegetables to maximize mental health benefits.

## Introduction

“You are what you eat” is a well-known adage that is increasingly supported by evidence linking healthy diets to optimal physical and mental health (Rooney et al., 2013; Robberecht et al., 2017). An important driver of the relationship between diet and health is high fruit and vegetable intake (FVI) (Lampe, 1999; Trichopoulou et al., 2003). Fruits and vegetables contain a variety of micronutrients critical to physical and mental function (Kaplan et al., 2007). Antioxidants such as vitamin C and carotenoids are said to play a pivotal role in protecting the body against oxidative stress, which is responsible for the causation and progression of neurodegenerative diseases, chronic inflammatory disease, atherosclerosis, some cancers, and some forms of depression (Byers and Perry, 1992; Irshad and Chaudhuri, 2002; Raison and Miller, 2011). Furthermore, the water-soluble vitamins (vitamin C, and B vitamins), and certain minerals (calcium, magnesium, and zinc), are important for optimal cognitive and emotional functioning (Huskisson et al., 2007; Kaplan et al., 2007).

There is now good evidence that higher FVI is related to better mental health. Research has established that people who eat more fruits and vegetables have a lower incidence of mental disorders, including lower rates of depression, perceived stress, and negative mood (Trichopoulou et al., 2003; Mikolajczyk et al., 2009; Jacka et al., 2010, 2011, 2017; Ford et al., 2013; Gopinath et al., 2016; Bishwajit et al., 2017; Li et al., 2017). People who eat more fruits and vegetables also have a higher likelihood of optimal mental states, such as greater happiness (Lesani et al., 2016), positive mood (Ford et al., 2013; White et al., 2013), life satisfaction (Blanchflower et al., 2013; Mujcic and Oswald, 2016), and socio-emotional flourishing, which captures feelings of meaning, purpose, and fulfillment in life (Conner et al., 2015, 2017a). Importantly, these associations between FVI and various mental health indicators appear to be (i) dose-dependent (to various points) whereby higher intakes of fruit and vegetables (FV) are associated with increasingly higher mental health scores (e.g., Blanchflower et al., 2013), (ii) robust when controlling for demographic, economic/social, and health covariates (e.g., gender, income, education, BMI, smoking, exercise; Blanchflower et al., 2013; Mujcic and Oswald, 2016; Bishwajit et al., 2017), and (iii) bolstered by longitudinal and intervention research that has shown causal relationships between higher FVI and mental health (Carr et al., 2013; Mujcic and Oswald, 2016; Conner et al., 2017a; Jacka et al., 2017). For example, using longitudinal data from 12, 389 people in the Household, Income, and Labor Dynamics in Australia (HILDA) Survey, Mujcic and Oswald (2016) found that a shift from “low” to “high” intake of FV across a period of 2 years resulted in significant improvement in life satisfaction, showing an average gain comparable to moving from unemployment to employment. Interventions have also shown that increasing fruit and/or vegetable consumption improves depressive symptoms among clinically-depressed adults (Jacka et al., 2017), improves feelings of vigor in young men with low baseline levels of vitamin C and a higher baseline mood disturbance (Carr et al., 2013), and increases flourishing in young adults with a low baseline consumption of FV (Conner et al., 2017a). Some research has indicated that positive mood states can also shift people toward healthier food choices (Gardner et al., 2014), and negative mood states such as stress can shift people toward unhealthier food choices and overeating (Singh, 2014); however, the longitudinal and experimental research designs outlined above provide convincing evidence that FVI can also have a direct and causal impact on subsequent psychological well-being.

While it is clear that there is a relationship between FVI and mental health, it is still unknown whether the ways that fruits and vegetables are prepared and consumed—that is, whether they are eaten raw, cooked, or from cans—might have distinctly different effects on mental health. There is evidence from the nutrition literature that the nutrient content in FV is reduced with cooking and canning. Cooking fruits and vegetables can alter the bioavailability of nutrients, which may have been hypothesized to play an influential role in the neurotransmission systems involved in mood and well-being (Kaplan et al., 2007). Water-soluble nutrients such as vitamin C and B vitamins are particularly vulnerable to heat degradation (Nicoli et al., 1999; Lee and Kader, 2000; Rickman et al., 2007a,b), which means that cooking would reduce the amount of mental health-conferring micronutrients from foods like spinach, bell peppers/capsicum, and green beans. Some evidence also suggests that cooking can reduce the quantity and activity of antioxidants (Nicoli et al., 1999; Zhang and Hamauzu, 2004), which is considered another mechanism linking FVI to mental health (Kaplan et al., 2007). However, other research indicates that cooking can actually *enhance* the bioavailability and activity of antioxidants (Dewanto et al., 2002; Turkmen et al., 2005; Miglio et al., 2008), and, that fat-soluble nutrients such as vitamins A, D, E, and K are less susceptible to damage by heat and processing than water-soluble nutrients, thus limiting the deleterious effects of cooking on nutrient profiles (Rickman et al., 2007b; Yuan et al., 2009). The effects of cooking on nutrient profiles may also differ between types of fruits and vegetables; for example, cooking tomatoes enhances the bioavailability of nutrients such as lycopene and antioxidants, whereas cooking broccoli loses many of its vital nutrients (Dewanto et al., 2002; Vallejo et al., 2002; Yuan et al., 2009). Further, eating canned FVI may also confer a nutrient profile similar to cooked FVI. Evidence has shown that longer storage times for canned fruits and vegetables can reduce the bioavailability of nutrients (Rickman et al., 2007a). Other forms of processing like freezing may not be as deleterious on nutrient content (Hebrero et al., 1988; Asami et al., 2003), but may depend on how people consume the frozen produce; for example, frozen berries eating in smoothies might retain their nutrient density, whereas frozen vegetables, which typically requires thawing and cooking, might have reduced nutrient content. Overall, the nutritional evidence regarding processing (cooking, canning, freezing), and nutrient loss in fruits and vegetables is nuanced. Yet for key micronutrients that have been linked to mental health such as vitamin C and carotenoids (Boehm et al., 2013; Carr et al., 2013), cooking and canning would most likely lead to a degradation in nutrients, thereby limiting their beneficial impact on mental health.

There is some evidence of differential associations with mental health depending on whether FV is consumed raw or in processed forms. We found three correlational studies that measured raw and cooked FVI separately and reported their associations with mental health; all three found that raw FVI was the stronger predictor of some (but not all) mental health outcomes than cooked FVI (Appleton et al., 2007; Mikolajczyk et al., 2009; El Ansari et al., 2014). In one study of 10,602 men living in France and Ireland, higher intakes of raw fruits and vegetables were significant predictors of less depressed mood (*b* = −0.10 and *b* = −0.13, respectively), while cooked vegetables were not (*b* = −0.02) (Appleton et al., 2007). In another a study of 3,706 undergraduate students across the United Kingdom, only consumption of salads/raw vegetables was negatively associated with stress in men; whereas raw fruit, raw vegetables, and cooked vegetables all significantly related to lower stress levels in women (El Ansari et al., 2014). Finally, in a study of 1,800 European students, there was a stronger inverse relationship between the consumption of salad and stress (*b* = −1.121) than that of cooked vegetables and stress (*b* = −0.82) in women; however, there were no differences between salads vs. cooked vegetables in regards to depressive symptoms (*b* = −1.69 vs. *b* = −1.69, both significant) (Mikolajczyk et al., 2009).

The differential effects of raw vs. processed FVI on mental health could help to explain the results of a recent intervention study by Conner et al. (2017a). In that study, participants were randomized to either receive fresh FV for 2 weeks (a parcel of fresh carrots, kiwifruit or oranges, and apples), receive daily text-message reminders to increase FVI for 2 weeks (plus given a voucher to purchase FV), or a diet-as-usual control group. Both the intervention groups reported significant increases in FVI; however, only the group that directly received FV reported improved well-being. The authors proposed that this difference might be accounted for by the nature in which the FV were consumed. The group who received fresh FV indicated they were more likely to consume their produce raw, while the reminder group indicated higher rates of cooked FV. Although Conner et al. (2017a) did not measure preparation and eating methods except informally through a retrospective questionnaire, they speculated that the ways in which their participants chose to eat their produce may have influenced the extent to which FVI affected their participants' well-being.

In spite of the preliminary evidence for the differential role of raw vs. processed FVI, it is still not completely clear whether consumption of raw FVI is superior to cooked or processed FVI in regards to mental health benefits. Although some preliminary evidence suggests an advantage of raw FVI over processed FVI (Appleton et al., 2007; Mikolajczyk et al., 2009; El Ansari et al., 2014; Conner et al., 2017a), research has not expressly tested the differential associations between raw vs. cooked/processed FVI on mental health outcomes, and, prior studies have largely been restricted to negative aspects of mental health such as depression (except Conner et al., 2017a).

The aim of the current study was to investigate whether raw FVI is more strongly associated with a range of mental health outcomes than processed FVI in a cross-sectional survey of over 400 young adults. We contrasted consumption of raw fruit and raw vegetables with relatively more processed forms of these foods (cooked, frozen, canned or tinned, as a group) in order to investigate the benefits of FV in an unmodified state (raw), compared to FV that has undergone a level of processing that may cause changes to the nutrient quality and quantity. Six aspects of mental health were measured to capture both negative and positive aspects of the illness-wellness continuum: depressive symptoms, anxiety, negative mood, positive mood, life satisfaction, and flourishing. A wide range of demographic and health covariates were also measured. It was hypothesized that stronger associations would occur between raw FVI and mental health than between cooked or processed FVI and mental health, and that these associations for raw FVI and mental health would remain significant when controlling for the covariates. It was also hypothesized that there would be stronger relationships between raw FVI and the presence of positive mental health rather than absence of negative mental health, given the patterns in recent literature. Overall, evidence of a stronger association between raw FVI and mental health outcomes (vs. cooked/processed FVI and mental health outcomes) could have implications for public health policy recommendations to consume more fruits and vegetables in their raw and unprocessed forms.

## Materials and methods

This was a cross-sectional, correlational design. Because fruit and vegetable consumption varies by age (Billson et al., 1999; University of Otago and Ministry of Health, 2011), our study focused on a single age group of young adults ages 18–25. Young adults typically have the lowest fruit and vegetable consumption of all age groups (Thompson et al., 1999; University of Otago and Ministry of Health, 2011) and they are at high risk for mental health disorders (Johnston et al., 2014).

### Participants and procedure

Table 1 presents the participant characteristics of the sample. The participants were 422 young adults between 18 and 25 years old. Participants were recruited either as part of their undergraduate psychology course at a large New Zealand university (*N* = 105), or through Amazon's Mechanical Turk (MTURK, *N* = 317) an online crowdsourcing marketplace that allows researchers to source large groups of people to complete online surveys in exchange for payment. Participants needed to be 18–25 years old, and MTurk participants were required to be living in the United States, Australia, or the United Kingdom due to similar dietary patterns to the local New Zealand sample allowing for ease of comparison. However, too few participants were recruited from Australia and the United Kingdom. As such, the final MTurk sample included only individuals from the United States. All participants were provided with an electronic information sheet about the questionnaire, which was broadly advertised as a questionnaire about lifestyle factors with no specific reference to the aims of investigating diet or mental health. This study was approved by the University of Otago Human Ethics Committee (Category B) (#D17/158) and all participants provided informed consent by way of electronic signature. Upon completing the 25-min online questionnaire, Psychology Students were remunerated with course credits for completing a brief worksheet based on their participation and MTURK participants received a small cash payment of US$1.50. MTURK participants were required to complete various attention checks embedded in the questionnaire to ensure accurate and meaningful answers were being obtained (*N* = 76 did not pass attention checks and were not allowed to continue with the survey). Data were collected between the months of March and June 2017.

**Table 1:** Descriptive Statistics for the Sample (*n* = 422).

	**% (*n*)**	**Mean (*SD*)**	**Min**	**Max**
Age		21.58 (2.12)	18	25
Gender				
Female	66.1 (279)			
Male	32.3 (136)			
Gender diverse	1.7 (7)			
Ethnicity				
Caucasian	67.5 (285)			
Asian	9.7 (41)			
Black	6.2 (26)			
Mixed	6.2 (26)			
Hispanic	5.2 (22)			
Other	5.2 (22)			
Sample				
% MTURK	75.12 (317)			
% Psychology Students	24.88 (105)			
% Full- or part-time students	65.6 (277)			
SES		4.12 (1.24)	1	7
BMI		24.85 (5.36)	14.7	53.78
Sleep quantity (hours/night)		7.27 (1.43)	2	20
Sleep quality		1.74 (1.01)	0	4
Days of physical activity/week		2.99 (1.95)	0	7
Alcohol (servings/week)		4.43 (6.74)	0	48
Smoker (Yes)	10.2 (43)			
Health condition (Yes)	25.6 (108)			
Food allergy (Yes)	16.6 (70)			
Antidepressant use (Yes)	15.9 (67)			
Supplement use (Yes)	35.8 (151)			
Vegetarian	10.0 (42)			
Food preparation				
Myself	43.8 (185)			
Roommates/in a group	17.5 (74)			
With partner	16.8 (71)			
Parents	10.2 (43)			
University dorm	10.0 (42)			
Other	1.7 (7)			
Raw FVI servings/day		2.20 (1.79)	0	12
Raw fruit		1.22 (1.13)	0	6
Raw vegetables		0.98 (0.99)	0	7
Processed FVI servings/day		0.99 (0.95)	0	5
Processed fruit		0.11 (0.33)	0	3.57
Processed vegetables		0.88 (0.86)	0	5
Unhealthy foods servings/day		1.45 (1.48)	0	8.57
Chocolate		0.47 (0.63)	0	7
Candy		0.18 (0.36)	0	2.86
French fries		0.30 (0.39)	0	2.86
Soda		0.49 (1.03)	0	7
Depressive symptoms		17.53 (11.95)	0	55
Anxiety symptoms		6.55 (4.43)	0	21
Negative mood		1.42 (0.78)	0	3.75
Positive mood		2.22 (0.82)	0	4
Life satisfaction		21.85 (7.26)	5	35
Flourishing		40.84 (9.29)	13	56

*BMI, body mass index; FVI, fruit and vegetable intake; MTurk, Mechanical Turk; SES, socioeconomic status*.

### Measures

#### Demographics

The first section of the questionnaire contained demographic covariate measures of age, gender (male, female, or gender diverse), ethnicity (Caucasian, Asian, Black, Hispanic, Mixed, other), student/employment status, and childhood and current socio-economic status (SES). Childhood SES was measured with three items (“My family usually had enough money for things when I was growing up;” “I grew up in a relatively wealthy neighborhood;” “I felt relatively wealthy compared to the other kids in my high school”); current SES was measured with three items (“I have enough money to buy things I want;” “I don't need to worry too much about paying my bills;” “I don't think I'll have to worry about money too much in the future”) (based on Griskevicius et al., 2011). Participants stated how much they agreed with each item using a Likert scale that ranged from 1 (Strongly Disagree) to 7 (Strongly Agree).

#### Lifestyle factors and other health behaviors

The second section contained a range of health and lifestyle covariate measures. Participants rated the quantity and quality of their sleep using two items from the Basic Nordic Sleep Questionnaire (Partinen and Gislason, 1995), asking “In a typical week, how many hours per night do you usually sleep?” and “How refreshed do you feel when waking up from sleep?” with five response options ranging from “Never refreshed” to “Very refreshed.” A single item was used to measure physical activity, asking how many days in a typical week an individual completes at least 30 min of exercise that was “enough to raise your breathing rate” (Milton et al., 2011). A number of examples of physical exercise were provided (e.g., cycling) as well as exclusions (e.g., housework). Participants entered their height and weight (responses available in both imperial and metric units) which was used to compute BMI (Nuttall, 2015). Participants indicated (yes/no) if they had any known health conditions from a list of 10 conditions including diabetes (Type 1 or Type 2), hypertension, history of cancer, osteoporosis, disordered eating behavior, cardiovascular disease, anemia, Chronic Fatigue Syndrome, Irritable Bowel Syndrome or Crohn's Disease, or “other,” and they indicated (yes/no) whether they had any food allergies from a list (i.e., dairy, eggs, peanuts, tree nut, wheat, soy, shellfish or fish, or other). Alcohol consumption was assessed by asking how many days in a typical week they consumed alcohol, and on those days when consuming alcohol, how many standard drinks they typically consumed, which were multiplied to derive a weekly alcohol consumption estimate. Participants also answered whether they currently used prescription anti-depressant or mood stabilizing medication, and whether they regularly took any vitamin or mineral supplements. Smoking status was assessed by asking how often participants smoked with five options: “I don't smoke now,” “Less than once a month,” “At least once a month,” “At least once a week,” and “At least once a day.”

#### Dietary assessment

The second section of the survey also contained a range of dietary assessment questions, but the four categories relevant to this report are: *raw vegetables*; *cooked/frozen/canned/tinned vegetables* (processed vegetables); *raw fruits*; and *cooked/canned/tinned fruits* (processed fruits). Table 2 lists the dietary questions. For each food category, participants estimated the number of days per week they ate that food (0–7 days/week). If they reported eating that food at least 1 day per week, then they reported the number of servings they typically consumed on days when they ate that food (1–7+ servings/day, serving defined for each food category) and the types of foods they typically consumed from a checklist of commonly consumed foods in that category (e.g., carrots, lettuce, etc.). These three questions provided quantitative information regarding the intake of various food groups and also more richly descriptive information about the types of foods they typically ate within each food category. This method of dietary assessment has been used in similar previous research (Lesani et al., 2016; Mujcic and Oswald, 2016). In addition, an index of four unhealthy foods (chocolate, candy/lollies, French fries/hot chips, and soda; see Table 2) was measured as a covariate as well as several other food groups not discussed in the present report (legumes, juices).

**Table 2:** Food survey questions (and response items).

**Raw Vegetables**
- How many days in a week do you eat raw vegetables? For example salads, carrots etc. Do not include vegetable juice. *[Response options: 0–7 days/week] If Response is >0 days then:* - On a day when you eat raw vegetables, how many servings of raw vegetables do you usually eat? 1 serving = 1 cup of salad or 1 large carrot *[Response options: 1–7+ servings]* - What types of raw vegetables do you usually eat? Please select all that apply. *[Response options: carrot, tomato, lettuce, spinach or other leafy greens e.g., kale, bok choy, silver beet, cucumber, cabbage, capsicum/bell pepper, beetroot/beets, celery, mushrooms, red onion, other (please specify)]*
**Processed Vegetables**
- How many days in a week do you eat cooked, frozen, or canned/tinned vegetables? For example vegetables cooked in a curry or stew; roast, boiled or steamed vegetables; canned/tinned tomatoes, green beans; frozen veggie mixes. (Do not include hot chips/French fries, kumara chips or deep fried potatoes; Do not include legumes such as baked beans, kidney beans, chick peas etc.). *[Response options: 0–7 days/week] If Response is >0 days then:* - On a day when you eat cooked, frozen, or canned/tinned vegetables, how many servings of cooked, frozen, or canned/tinned vegetables do you usually eat? 1 serving = ½ cup of cooked or frozen vegetables or ½ tin of canned vegetables etc. *[Response options: 1–7+ servings]* - What types of cooked, frozen, or canned/tinned vegetables do you usually eat? Please select all that apply. *[Response options: potato, kumara, carrots, tomatoes (canned/tinned), tomatoes (cooked), onions/leeks, corns, cauliflower, broccoli, asparagus, zucchini/courgette, eggplant/aubergine, beetroot/beets, mushroom, pumpkin, green beans, capsicum or bell pepper, spinach or other leafy greens e.g., kale, bok choy, silver beet, mixed frozen vegetables, other (please specify)]*
**Raw Fruit**
- How many days in a typical week do you eat raw fruits? For example banana, apple, orange, kiwi fruit, berries. Please include any frozen fruit if eaten raw (such as in smoothies). Do not include fruit juice or dried fruit. *[Response options: 0–7 days/week] If Response is >0 days then:* - On a day when you eat raw fruits, how many servings of raw fruits do you usually eat? 1 serving = 1 apple or 1 banana or 2 kiwifruit or 1/2 cup of berries (fresh or frozen) *[Response options: 1–7+ servings]* - What types of raw fruits do you usually eat? Please select all that apply. *[Response options: banana, apple, orange, mandarin, tangerine, stone fruit (peach, nectarine, apricot, plum), pear, berries (fresh), berries (frozen but eaten raw), kiwi fruit, grapes, grapefruit, other (please specify)]*
**Processed Fruit**
- How many days in a typical week do you eat cooked or canned/tinned fruits? For example stewed apple or tinned peaches *[Response options: 0–7 days/week] If Response is >0 days then:* - On a day when you eat cooked or canned/tinned fruits, how many servings of cooked or canned/tinned fruits do you usually eat? 1 serving = ½ cup of cooked fruits or ½ tin of canned fruits etc. *[Response options: 1–7+ servings]* - What types of cooked canned/tinned fruits do you usually eat? Please select all that apply. *[Response options: peaches, apricots, plums, pears, berries, apples, pineapple, other (please specify)]*
**Chocolate**
- How many days in a week do you eat chocolate? (e.g., chocolate bars or blocks, chocolate slices or baked goods, chocolate desserts, including ice cream) *[Response options: 0–7 days/week] If response is >0 days then:* - On a day when you eat chocolate, how many servings of chocolate do you eat? 1 serving = one medium chocolate bar or 5 squares of chocolate from a large block *[Response options: 1–7+ servings]*
**Candy**
- How many days in a typical week do you eat candy or lollies? For example fruit bursts, gummy bears, sour snakes, liquorice, jubes, barley sugars. Do not include chocolate candies. *[Response options: 0–7 days/week] If response is >0 days then:* - On a day when you eat candy or lollies, how many servings of candy or lollies do you eat? 1 serving = ½ handful of gummy bears or 5 fruit bursts or 1 medium stick of liquorice *[Response options: 1–7+ servings]*
**French Fries**
- How many days in a typical week do you eat hot chips, French fries, kumara chips, or wedges? *[Response options: 0–7 days/week] If response is >0 days then:* - On a day when you eat hot chips, French fries, kumara chips or wedges, how many servings of *hot chips, French fries, kumara chips, or wedges* do you eat? 1 serving = one cup or 1 small/regular fast food serving or ½ scoop of takeaway hot chips. *[Response options: 1–7+ servings]*
**Soda**
- How many days in a typical week do you drink soda? (Include diet or low calories types) *[Response options: 0–7 days/week] If response is >0 days then:* - On a day when you drink soda, how many servings of soda do you drink? 1 serving = 250 mL *[Response options: 1–7+ servings]*

Following the dietary assessment, participants were asked additional questions about their dietary habits including how they typically purchase and prepare their food with six possible responses (“I mainly purchase and prepare my food myself,” “I mainly buy and cook food as a flat, apartment, or in a group,” “My parents mainly prepare my food” or “I mainly prepare and purchase my food with my partner,” “I mainly eat at my University Residence Hall/Dormitory,” or “other”), and, whether they restrict or exclude certain foods based on health or ethical reasons to measure vegetarian status.

### Mental health measures

#### Depressive symptoms

Depressive symptoms were measured using the Centre for Epidemiological Depression Scale (CESD; Radloff, 1977). Participants rated 20 statements about feelings of depression “in the last week including today,” with the response options “Rarely or none of the time (<1 day),” “Some or a little of the time (1–2 days),” “Occasionally or a moderate amount of the time (3–4 days),” and “Most or all of the time (5–7 days),” corresponding to an item score of 0, 1, 2, or 3. Responses were summed, reverse scoring as needed (α = 0.929).

#### Anxiety

Anxiety was measured using the 7 item Hospital Anxiety and Depression Scale—Anxiety Subscale (HADS-A; Zigmond and Snaith, 1983). Anxiety symptoms felt “in the last week including today,” were rated with response options of “Not at all,” “From time to time, occasionally,” “A lot of the time,” and “Most of the time,” corresponding to an item score of 0, 1, 2, or 3. Responses were summed, reverse scoring as needed (α = 0.854).

#### Negative and positive mood

Negative and positive mood was measured using a scale based on the affective circumplex (Barrett and Russell, 1999). The scale consisted of 24 mood items that varied by valence (negative/positive) and activation (high/medium/low). The negative mood items were *hostile, stressed, irritable, angry, anxious, annoyed, nervous, tense, hopeless, unhappy, dejected*, and *sad*. The positive mood items were *enthusiastic, excited, energetic, joyful, happy, cheerful, pleasant, good, relaxed, calm, content*, and *satisfied*. Items were randomized and presented in the same order for all participants. Participants responded to the question “Typically, do you feel…” for each item using a Likert scale anchored at 0 (None of the time), 1 (A little of the time), 2 (Some of the time), 3 (A good bit of the time), and 4 (Most of the time). Responses were averaged for a measure of negative mood (α = 0.945) and positive mood (α = 0.953).

#### Life satisfaction

Life Satisfaction was measured with the Satisfaction with Life Scale (SWLS; Diener et al., 1985). Participants rated five statements for how they “personally feel at this time in [their] life,” e.g., “In most ways, my life is close to ideal” and “If I could live my life over, I would change almost nothing.” Responses were made on a Likert scale from 1 (Strongly disagree) to 7 (Strongly agree), which were summed (α = 0.905).

#### Flourishing

Flourishing was measured with the Flourishing Scale (Diener et al., 2010). Participants rated their agreement with eight statements related to well-being, including “I am engaged and interested in my daily activities” and “I lead a purposeful and meaningful life” on a Likert scale from 1 (Strongly disagree) to 7 (Strongly agree), which were summed (α = 0.922).

### Data preparation

Six participants were excluded due to incomplete data and two participants were excluded due to suspected errors in responding, which resulted in a final sample size of 422 participants. Gender was dummy coded using two variables with male gender as the reference group (male, female, and gender diverse coded as 0, 1, 0, and 0, 0, 1, respectively). Ethnicity was dummy coded using three variables with Caucasian ethnicity as the reference group (Caucasian, Asian, Black, all others, as 0, 1, 0, 0, and 0, 0, 1, 0, and 0, 0, 0, 1), respectively. Student status was dummy coded as 0 for non-students and 1 for any full- or part-time students. Childhood and current SES items were averaged to produce a total SES score (α = 0.844). BMI was computed by dividing weight by kilograms by the square of height in meters (Nuttall, 2015). Smoking was dummy coded to indicate non/infrequent smokers (0) vs. regular smokers who smoked at least once per week or more (1). Health condition, food allergy, and food restriction variables were dummy coded to indicate absence (0) or presence (1) of any major health condition, food allergy, or vegetarianism, respectively.

Average daily servings of food groups were calculated by multiplying days per week consumed by servings per day consumed, and then dividing by seven to get an average daily intake estimate. This computation was done for each food category separately. We also created a combined raw fruit and vegetable daily intake (raw FVI) variable by summing the daily raw fruit and daily raw vegetable serving estimates together, and a combined processed fruit and vegetable intake (processed FVI) variable by summing the daily cooked/canned fruit and daily cooked/canned/frozen vegetable estimates together. Lastly, we created a combined unhealthy food index by summing the daily servings for chocolate, candy, French fries, and soda, however, due to low reliability (α = 0.276), the items were analyzed separately.

### Statistical analyses

Firstly, between-person (cross-sectional) relationships were tested using bivariate correlation coefficients in SPSS to investigate whether average raw and processed FVI, as well as unhealthy food intake, were associated with mental health outcomes.

Secondly, a series of hierarchical regression analyses were conducted to predict the six mental health outcomes—depressive symptoms, anxiety, negative mood, positive mood, life satisfaction, and flourishing—from raw FVI and processed FVI as simultaneous predictors, controlling for the demographic and health covariates. All continuous variables were centered for analysis. In the first step, we entered the two fruit and vegetable variables (raw FVI and processed FVI, both centered) as simultaneous predictors plus their quadratic terms to test for any non-linear associations with the mental health outcomes. Non-significant quadratic terms were dropped from the final models for simplicity. In the second step, we entered covariates to isolate the unique associations between FVI and mental health. Covariates were included in the model if they correlated with either the predictors (raw FVI or processed FVI) and/or any of the mental health outcome measures. The covariate related to food preparation was dummy coded 1 if their parents prepared their food and 0 for all others because this was the only contrast that covaried with the predictor(s)/outcome(s).

Lastly, we conducted exploratory analyses to determine which individual types of raw or processed fruits and vegetables were most strongly associated with mental health. Unadjusted bivariate correlations were computed between endorsement of a given food (0 vs. 1) and each of the six mental health measures to investigate whether particular food items were more strongly related to mental health outcomes than others.

## Results

### Descriptive data

The descriptive statistics can be found in Table 1. Overall, the sample was predominantly female (66.1%) and a majority identified as Caucasian (67.5%). The combined childhood and adult mean SES score (4.12) fell within a middle range, suggesting that participants perceived themselves as no better or worse off than others. The average BMI (24.85) was at the higher end of a healthy range (18–25). In regards to food consumption, healthy food items (fruits, vegetables) were eaten more frequently than the unhealthy foods. Participants reported eating ~3.2 daily servings of FV, which mostly consisted of raw fruit (1.2 servings), raw vegetables (1.0 servings), and processed vegetables (0.9 servings). Processed fruits were not frequently eaten (0.1 servings). The mean depressive symptoms score was 1.5 points above the 16-point cut-off for CES-D scores indicating possible risk for clinical depression. The mean anxiety score was below the 8-point cut-off on the HADS-A and therefore indicative of normal levels of anxiety. Positive mood was higher than negative mood, but life satisfaction was at the lower end of the average range for American college students (20–24) (Pavot and Diener, 1993) and flourishing was in the lower 25% compared to American college students (Diener et al., 2010).

There were several significant differences between the MTURK and Psychology participants (data available from authors). Those recruited from MTURK (vs. Psychology) tended to be more male (36.0 vs. 21.0%), more ethnically diverse (66.6 vs. 70.5% Caucasian), older age (22.3 vs. 19.5 years), lower SES (4.0 vs. 4.6), have greater BMI (25.38 vs. 23.25), and feel less satisfied with their lives (21.2 vs. 23.7). Because of this difference, sample was included as a covariate in the regression analyses (coded Psychology = 0; MTurk = 1).

Table 3 presents the inter-correlations among the fruit and vegetable measures, the unhealthy foods measures, and the mental health measures. The correlation between raw FVI and processed FVI was 0.265, *p* < 0.001. The correlation between raw fruits and raw vegetables was 0.422, *p* < 0.001. The correlation between processed fruits and processed vegetables was 0.103, *p* < 0.05. There were few associations between the fruit and vegetable measures and unhealthy foods. Consumption of processed FVI, particularly processed fruits, was associated with more unhealthy foods like candy and French fries. The unhealthy foods correlated with each other, with the exception of chocolate. All of the mental health measures were significantly correlated with each other above |*r*| 0.50, all *p*s < 0.001.

**Table 3:** Inter-correlations among the fruit, vegetables, unhealthy foods, and mental health measures.

	**Raw FVI**	**Raw fruits**	**Raw vegetables**	**Processed FVI**	**Processed fruits**	**Processed vegetables**
Raw FVI	1	0.866^***^	0.819^***^	0.265^***^	0.156^**^	0.234^***^
Raw fruits		1	0.422^***^	0.232^***^	0.133^**^	0.206^***^
Raw vegetables			1	0.215^***^	0.130^**^	0.188^***^
Processed FVI				1	0.434^***^	0.941^**^
Processed fruits					1	0.103^*^
Processed vegetables						1
	**Unhealthy foods**	**Chocolate**	**Candy**	**French fries**	**Soda**	
Raw FVI	−0.053	−0.007	0.043	0.013	−0.092	
Raw fruits	−0.024	−0.009	0.064	0.047	−0.070	
Raw vegetables	−0.068	−0.002	0.004	−0.031	−0.087	
Processed FVI	0.089	0.073	0.169^***^	0.103^*^	−0.014	
Processed fruits	0.144^**^	0.044	0.280^***^	0.194^***^	0.008	
Processed vegetables	0.044	0.064	0.081	0.041	−0.019	
Unhealthy foods	1	0.502^***^	0.512^***^	0.486^***^	0.767^***^	
Chocolate		1	0.236^***^	0.040	0.015	
Candy			1	0.380^***^	0.098^*^	
French fries				1	0.159^**^	
Soda					1	
	**Depressive symptoms**	**Anxiety**	**Negative mood**	**Positive mood**	**Life satisfaction**	**Flourishing**
Depressive symptoms	1	0.747^***^	0.749^***^	−0.710^***^	−0.642^***^	−0.667^***^
Anxiety		1	0.734^***^	−0.532^***^	−0.496^***^	−0.465^***^
Negative mood			1	−0.569^***^	−0.566^***^	−0.566^***^
Positive mood				1	0.684^***^	0.777^***^
Life satisfaction					1	0.719^***^
Flourishing						1

*FVI, Fruit and vegetable intake*;

* *p < 0.05*;

** *p < 0.01*;

*** *p < 0.001*.

### Bivariate correlations between fruit and vegetable intake and mental health, without adjustment for covariates

The bivariate correlations between FVI and measures of mental health are presented in Table 4. Correlations between the unhealthy foods and mental health are also presented for completeness. Raw fruits and vegetables had the strongest associations with most of the mental health measures. Raw FVI was associated with fewer depressive symptoms and higher positive mood, life satisfaction, and flourishing. Raw fruits were additionally associated with reduced negative mood. By contrast, processed FVI was only associated with positive mood but not with any of the other mental health variables. The size of the correlations was significantly stronger for raw FVI than processed FVI for depressive symptoms (*Z* = −3.065, *p* = 0.001 one tailed), positive mood (*Z* = 1.912, *p* = 0.028 one tailed), life satisfaction (*Z* = 2.351, *p* = 0.009 one tailed), and flourishing (*Z* = 2.879, *p* = 0.002 one tailed) using the difference test between two dependent correlations with one variable in common (Lee and Preacher, 2013). The coefficients did not differ for anxiety (*Z* = −1.573, *p* = 0.058 one tailed) or negative mood (*Z* = −1.456, *p* = 0.073 one tailed). The unhealthy foods composite index was not related to the mental health variables, although higher soda consumption was correlated with more depressive symptoms and lower life satisfaction.

**Table 4:** Bivariate correlations between intake of fruit, vegetables, unhealthy foods, and mental health.

	**Depressive symptoms**	**Anxiety symptoms**	**Negative mood**	**Positive mood**	**Life satisfaction**	**Flourishing**
Raw FVI	−0.157^**^	−0.077	−0.090	0.280^**^	0.161^**^	0.205^**^
Raw fruits	−0.161^**^	−0.092	−0.113^*^	0.279^**^	0.157^**^	0.218^**^
Raw vegetables	−0.100^*^	−0.035	−0.034	0.188^***^	0.112^*^	0.122^*^
Processed FVI	0.023	−0.016	−0.004	0.171^**^	0.023	0.037
Processed fruits	0.002	−0.014	−0.023	0.106^*^	0.045	−0.008
Processed vegetables	0.025	−0.013	0.004	0.149^**^	0.008	0.044
Unhealthy foods	0.068	0.055	0.051	−0.004	−0.072	−0.040
Chocolate	0.019	−0.005	0.048	0.044	0.046	0.012
Candy	0.027	0.001	0.013	0.084	0.034	0.040
French fries	−0.056	−0.028	−0.044	0.088	0.003	0.028
Soda	0.098^*^	0.093	0.056	−0.095	−0.144^**^	−0.090

*FVI, Fruit and vegetable intake*;

* *p < 0.05*;

** *p < 0.01*;

*** *p < 0.001*.

### Regression models using fruit and vegetable intake to predict mental health, adjusting for covariates

**Figure 1:**
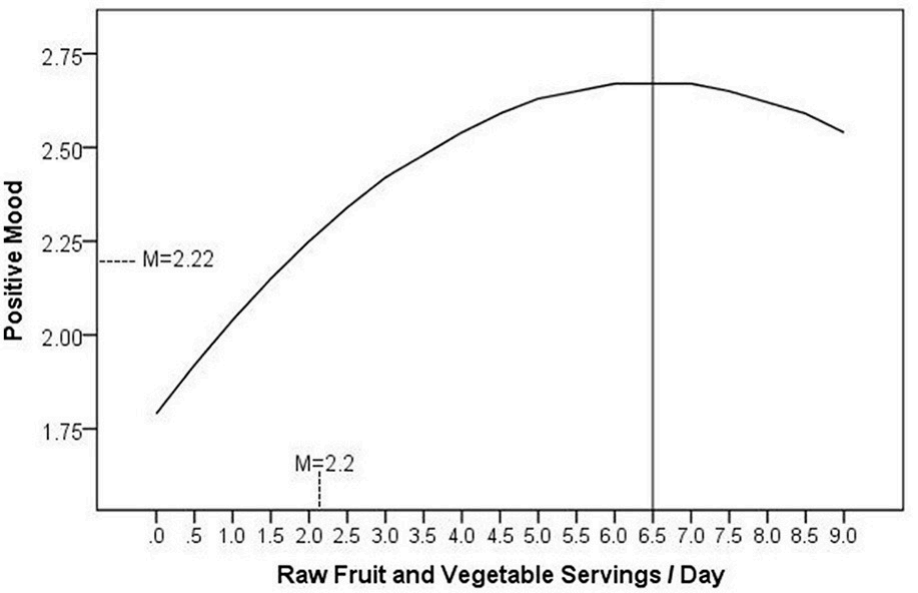
The curvilinear relationship between consumption of raw fruits and vegetables and positive mood, adjusted for covariates.

Results from the regression analyses are presented in Table 5. Raw FVI, but not processed FVI, significantly predicted lower depressive symptoms and higher positive mood, life satisfaction, and flourishing when controlling for the covariates. There was also a significant quadratic pattern between raw FVI and positive mood, as shown in Figure 1. The inflection point occurred at 6.5 servings per day. This indicates that incremental improvements in positive mood were observed up to six and a half servings of raw FVI a day, after which increasing servings of raw FVI was associated with no additional benefits to positive mood. A quadratic regression term predicting depression from raw FVI was significant in the first step, but was no longer significant when demographic and lifestyle covariates were included. Lastly, it is notable that raw FVI predicted all three of the positive mental health measures (positive mood, life satisfaction, and flourishing) and only one of the negative mental health measures (depressive symptoms), which is consistent with predictions that FVI will be more strongly related to positive than negative mental health.

**Table 5:** Regression models predicting mental health variables from fruit and vegetable intake, demographic covariates, and health related covariates.

**Predictors**	**Depressive symptoms B (SE)**	**Anxiety symptoms B (SE)**	**Negative mood B (SE)**	**Positive mood B (SE)**	**Life satisfaction B (SE)**	**Flourishing B (SE)**
Intercept	16.788(0.662)^***^	6.547(0.216)^***^	1.421(0.038)^***^	2.296(0.043)^***^	21.846(0.350)^***^	40.839(0.443)^***^
Raw fruits and vegetables	−1.819(0.442)^***^	−0.193(0.125)	−0.041(0.022)	0.185(0.029)^***^	0.675(0.203)^**^	1.090(0.257)^***^
Raw fruits and vegetables (Quad)	0.231(0.104)^*^	–	–	−0.025(0.007)^***^	–	–
Processed fruits and vegetables	0.951(0.623)	0.020(0.235)	0.017(0.041)	0.080(0.041)^*^	−0.161(0.380)	−0.179(0.428)
*R*^2^ Change	0.040^**^	0.006	0.009	0.116^***^	0.026^***^	0.042^***^
*F* Change (*df*)	*F*_(3, 418)_ = 5.87	*F*_(2, 419)_ = 1.251	*F*_(2, 419)_ = 1.805	*F*_(3, 418)_ = 18.368	*F*_(2, 419)_ = 5.658	*F*_(2, 419)_ = 9.293
*R*^2^ change controlling for covariates	0.030^**^	0.004	0.007	0.044^***^	0.013^*^	0.019^**^
*F* Change (*df*) controlling for covariates	*F*_(3, 397)_ = 6.418	*F*_(2, 398)_ = 1.189	*F*_(2, 398)_ = 1.862	*F*_(3, 397)_ = 10.66	*F*_(2, 398)_ = 4.16	*F*_(2, 398)_ = 5.15
Intercept	14.491(1.782)^***^	4.677(0.699)^***^	1.243(0.125)^***^	2.30(0.114)^***^	21.88(1.06)^***^	40.13(1.47)^***^
Raw fruits and vegetables	−1.522(0.395)^***^	−0.174(0.119)	−0.040(0.021)	0.135(0.025)^***^	0.476(0.181)^**^	0.787(0.251)^**^
Raw fruits and vegetables (Quad)	0.150(0.089)	–	–	−0.016(0.006)^**^	–	–
Processed fruits and vegetables	1.39(0.546)^*^	0.167(0.216)	0.033(0.039)	0.017(0.035)	−0.558(0.329)	−0.581(0.455)
Age	0.270(0.303)	−0.005(0.120)	0.002(0.022)	−0.043(0.019)^*^	−0.012(0.183)	−0.446(0.253)
Gender D1	−0.141(1.14)	0.723(0.451)	0.067(0.081)	0.125(0.073)	1.13(0.686)	2.09(0.948)^*^
Gender D2	8.29(3.98)^**^	3.16(1.58)^*^	0.639(0.283)^*^	−0.432(0.254)	−4.00(2.40)	−5.93(3.31)
Sample	0.487(1.49)	−0.797(0.591)	0.070(0.106)	−0.088(0.096)	−0.518(0.900)	−0.742(1.24)
Student	1.04(1.19)	0.425(0.472)	0.058(0.085)	−0.017(0.076)	−0.826(0.717)	−0.157(0.991)
SES	−2.04(0.425)^***^	−0.746(0.169)^***^	−0.104(0.030)^**^	0.171(0.027)^***^	2.76(0.257)^***^	1.77(0.355)^***^
BMI	0.136(0.096)	0.004(0.038)	0.002(0.007)	−0.003(0.006)	−0.059(0.058)	−0.043(0.080)
Sleep quantity	−0.057(0.359)	−0.093(0.142)	0.008(0.026)	0.033(0.023)	−0.122(0.217)	0.328(0.299)
Sleep quality	−4.06(0.541)^***^	−1.19(0.214)^***^	−0.231(0.038)^***^	0.290(0.035)^***^	1.32(0.236)^***^	2.11(0.451)^***^
Physical activity	−0.076(0.273)	−0.030(0.108)	−0.004(0.019)	0.020(0.017)	0.030(0.164)	0.313(0.226)
Alcohol	0.090(0.074)	0.034(0.029)	0.009(0.005)	.000(0.005)	−0.034(0.045)	−0.069(0.062)
Smoking	0.874(1.625)	0.299(0.644)	−0.109(0.116)	−0.023(0.104)	−0.253(0.980)	−1.10(1.35)
Health condition	1.48(1.19)	0.029(0.470)	0.029(0.084)	−0.026(0.076)	0.573(0.715)	0.788(0.988)
Food allergy	0.214(1.34)	0.345(0.529)	0.043(0.095)	−0.001(0.086)	0.144(0.805)	−0.376(1.11)
Antidepressant use	6.34(1.41)^***^	2.49(0.558)^***^	0.403(0.100)	−0.291(0.090)^*^	−1.34(0.849)	−3.03(1.17)
Supplement use	0.268(1.04)	0.052(0.411)	−0.028(0.074)	0.075(0.066)	0.381(0.626)	1.01(0.864)
Vegetarian	1.16(1.71)	0.538(0.674)	−0.006(0.121)	−0.032(0.109)	0.797(1.03)	0.158(1.42)
Parents prepare food	−2.42(1.63)	−0.949(0.648)	−0.226(0.116)	−0.002(0.104)	0.606(0.985)	1.54(1.36)
Candy	2.40(1.49)	0.256(0.591)	0.084(0.106)	0.005(0.095)	0.164(0.900)	0.381(1.24)
French fries	−2.93(1.36)^*^	−0.481(0.538)	−0.107(0.096)	0.189(0.087)^*^	0.299(0.818)	1.11(1.13)
Soda	−0.045(0.506)	0.105(0.201)	−0.004(0.036)	0.010(0.032)	−0.343(0.305)	0.120(0.422)
*R*^2^ Change	0.334^***^	0.278^***^	0.239^***^	0.338^***^	0.355^***^	0.236^***^
*F* Change (*df*)	*F*_(21, 397)_ = 10.07	*F*_(21, 398)_ = 7.344	*F*_(21, 398)_ = 6.004	*F*_(21, 397)_ = 11.692	*F*_(21, 398)_ = 10.879	*F*_(21, 398)_ = 6.189
*R* Square (All variables)	0.374	0.284	0.247	0.454	0.381	0.278

*SE, standard error; SES, socioeconomic status; all continuous variables are centered*.

* *p < 0.05*;

** *p < 0.01*;

*** *p < 0.001*.

### Single food item analyses

The unadjusted bivariate correlations between endorsement of a given food and each of the six mental health measures are presented in Tables 6, 7. In terms of raw vegetables, what could be considered “salad fixings” were most significantly related to aspects of mental health. These included vegetables like carrots, dark leafy greens (kale, spinach), lettuce, cucumber, red onion, cabbage, celery, tomato, and mushrooms. In terms of processed vegetables, pumpkin, mixed frozen vegetables, potatoes/sweet potatoes, broccoli, and eggplant were significantly related to positive mood, and several of these were also related to flourishing. Raw bananas and apples were the strongest predictors of most mental health measures and almost all other raw fruits such as grapefruit, berries, kiwifruit, stone fruit (peaches, apricots), pear, frozen berries (eaten raw), and grapes were related to positive mood and flourishing.

**Table 6:** Correlations between types of raw and processed vegetables, and mental health.

	**Depressive symptoms**	**Anxiety symptoms**	**Negative mood**	**Positive mood**	**Life satisfaction**	**Flourishing**
**RAW VEGETABLES**						
Carrot	−0.130^**^	−0.085	−0.108^*^	0.248^**^	0.187^**^	0.223^**^
Dark leafy greens	−0.145^**^	−0.077	−0.077	0.175^**^	0.197^**^	0.184^**^
Lettuce	−0.118^*^	−0.065	−0.04	0.204^**^	0.113^*^	0.174^**^
Cucumber	−0.076	−0.017	−0.037	0.186^**^	0.071	0.146^**^
Red onion	−0.088	−0.032	−0.043	0.156^**^	0.103^*^	0.152^**^
Cabbage	−0.072	−0.103^*^	−0.084	0.144^**^	0.064	0.090
Celery	−0.060	0.008	−0.007	0.137^**^	0.065	0.117^*^
Tomato	−0.017	0.019	−0.039	0.117^*^	0.071	0.078
Mushroom	−0.106^*^	−0.048	−0.057	0.108^*^	0.045	0.053
Bell pepper/Capsicum	−0.042	−0.002	−0.027	0.089	0.057	0.107^*^
Beets/Beetroot	−0.015	−0.022	−0.031	0.070	0.041	0.039
Other	0.068	0.094	0.091	−0.062	−0.017	−0.047
**PROCESSED VEGETABLES**						
Pumpkin	−0.020	−0.039	0.014	0.136^**^	0.091	0.081
Mixed frozen vegetables	−0.035	0.024	0.026	0.126^**^	0.037	0.113^*^
Potatoes	−0.029	0.021	0.018	0.116^*^	0.016	0.013
Broccoli	−0.065	−0.036	−0.037	0.110^*^	0.042	0.118^*^
Sweet potato/Kumara	−0.067	−0.057	0.035	0.115^*^	0.069	0.032
Asparagus	−0.096^*^	−0.052	−0.037	0.079	0.144^**^	0.086
Onions/leeks	0.058	0.080	0.119^*^	0.027	0.012	−0.019
Green beans	−0.032	0.037	−0.042	0.071	−0.045	0.102^*^
Eggplant/Aubergine	−0.049	−0.016	−0.024	0.099^*^	0.078	0.084
Dark leafy greens	−0.081	−0.081	−0.068	0.095	0.061	0.096^*^
Mushroom	−0.008	0.026	0.027	0.084	−0.038	0.010
Carrots	0.017	0.057	0.066	0.083	0.067	0.023
Cauliflower	−0.035	−0.028	0.024	0.080	0.065	0.025
Bell pepper/Capsicum	−0.031	−0.011	0.045	0.075	0.059	0.078
Tomatoes (canned)	0.021	0.043	−0.005	0.071	0.069	0.073
Tomatoes (cooked)	0.006	0.040	0.006	0.067	0.071	0.062
Beet/Beetroot	−0.035	−0.013	0.001	0.067	0.038	0.042
Zucchini/Courgette	−0.057	−0.041	−0.011	0.065	0.076	0.089
Corn	−0.035	0.020	0.023	0.036	−0.061	0.045
Other	0.037	−0.022	0.019	−0.051	−0.015	−0.045

*Processed, cooked/canned/frozen*;

* *p < 0.05*;

** *p < 0.01*.

**Table 7:** Correlations between types of raw and processed fruits, and mental health.

	**Depressive symptoms**	**Anxiety symptoms**	**Negative mood**	**Positive mood**	**Life satisfaction**	**Flourishing**
**RAW FRUIT**						
Banana	−0.138^**^	−0.078	−0.117^*^	0.224^**^	0.175^**^	0.242^**^
Apple	−0.152^**^	−0.119^*^	−0.114^*^	0.223^**^	0.173^**^	0.225^**^
Grapefruit	−0.065	−0.034	−0.054	0.208^**^	0.148^**^	0.149^**^
Citrus fruit	−0.091	−0.116^*^	−0.045	0.186^**^	0.093	0.194^**^
Berries (fresh)	−0.140^**^	−0.049	−0.075	0.176^**^	0.087	0.171^**^
Kiwi fruit	−0.073	−0.048	−0.082	0.146^**^	0.082	0.157^**^
Stone fruit	−0.035	0.034	0.020	0.136^**^	0.046	0.129^**^
Pear	0.006	0.034	0.044	0.115^*^	0.070	0.103^*^
Frozen berries (eaten raw)	−0.052	0.002	0.039	0.113^*^	0.088	0.125^**^
Grapes	−0.050	0.007	−0.041	0.100^*^	0.084	0.163^**^
Other	−0.042	−0.010	−0.026	0.027	0.049	0.018
**PROCESSED FRUIT**						
Peaches	−0.044	−0.053	−0.033	0.118^*^	0.045	0.098^*^
Pineapple	−0.020	−0.003	−0.009	0.098^*^	0.003	0.100^*^
Apples	−0.080	−0.085	−0.076	0.102^*^	0.045	0.089
Apricot	0.017	0.004	−0.013	0.096^*^	0.036	0.026
Plum	0.017	0.014	0.026	0.052	0.038	−0.027
Pear	0.020	0.027	0.033	0.047	−0.001	0.044
Berries	−0.021	−0.029	−0.021	0.041	0.068	−0.005
Other	−0.066	0.004	−0.014	0.002	−0.005	0.018

*Processed, cooked/canned/frozen*;

* *p < 0.05*;

** *p < 0.01*.

## Discussion

This study provided evidence that the consumption of raw fruits and vegetables has a stronger relationship with mental health than the consumption of cooked or canned (processed) fruits and vegetables. Although this was only a correlational design, the patterns were robust when controlling for demographic and health covariates, and they paralleled the findings of recent intervention research showing significant effects of increasing fresh FVI on mental well-being (e.g., through kiwifruit, Carr et al., 2013; through carrots, kiwifruit/oranges, apples, Conner et al., 2017a). Our study findings add to this literature by showing that when tested side-by-side, the consumption of raw fruits and vegetables significantly outperformed more processed forms of FVI in the prediction of mental health. While we did not test the mechanisms that may explain the stronger link between raw FVI and mental health, a likely factor is that raw fruits and vegetables deliver a greater amount of nutrients than cooked or canned fruits and vegetables. This idea is somewhat supported by the literature, with evidence indicating that cooking, canning, and processing can significantly decrease the nutrient content of some forms of produce (Nicoli et al., 1999; Lee and Kader, 2000; Zhang and Hamauzu, 2004; Rickman et al., 2007a). Raw fruits and vegetables may provide greater levels of micronutrients than processed fruits and vegetables, which could explain their stronger association with improved mental well-being. However, as outlined previously, evidence of significantly diminished nutrient levels in cooked and processed produce is varied, and appears to be individualized from nutrient to nutrient (Dewanto et al., 2002; Turkmen et al., 2005; Miglio et al., 2008). Further research is required to determine how processing affects nutrient levels in fruit and vegetables, and whether this actually translates to biologically significant differences in subsequent levels of micronutrients that are provided to the body.

Findings have several applications for the promotion of health and well-being. First, future experimental research should examine the effects of increasing raw fruit and vegetables on mental well-being, given that greater mood-conferring benefits are likely to be seen with a predominantly raw based fruit and vegetable supplementation program. If our patterns are confirmed in intervention studies, it would suggest that heath policies could focus on promoting the consumption of raw and unprocessed produce for optimal well-being. Such interventions will require educating individuals on ways to prepare and consume fruits and vegetables that are likely to retain the greatest levels of nutrients. Furthermore, there may be additional barriers in developing raw plant food interventions. Previous qualitative research has shown that people view raw produce as less convenient due to its perishable nature than canned/processed/frozen produce (Brug et al., 1995; Hartman et al., 2013). Another limiting factor is cost and availability. Research has also suggested that people eat raw fruits and vegetables as snacks, whereas people incorporate more cooked/canned/processed produce into main meals (Brookie et al., 2017), and that sometimes raw fruits and vegetables are not considered satiating enough for a main meal (Brug et al., 1995; Hartman et al., 2013). Moreover, some people are less open to eating plant foods (i.e., people low in openness to experience, Conner et al., 2017b). As such, any policy or intervention aimed at increasing the consumption of raw fruits and vegetables may benefit from addressing accessibility and affordability, considering variation in food preferences, increasing healthy snacking on fruits and vegetables (with a high likelihood that these will be raw), and highlighting the ways in which raw FV can be incorporated into main meals that are satiating and fulfilling.

Given the correlational design, we cannot be sure that food consumption is directly and causally driving improvements in mental health. As mentioned previously, mood states (both positive and negative) have the ability to influence subsequent food choices. However, the preliminary results achieved in the current study mean that more controlled experimental research—that would investigate directionality—is warranted. This study had other limitations aside from the correlational design. We used a non-validated food recall measure. However, this was based on previously designed and published measures suited to larger population studies (e.g., Lesani et al., 2016; Mujcic and Oswald, 2016). Additionally, there are inherent limitations associated with this type of dietary recall, including possible errors in estimation and memory recall, as well as inaccuracy in estimating serving sizes (Thompson and Byers, 1994). Future research should consider using gold standard methods such as a weighed dietary record.

Finally, the current study did not measure some additional factors that could influence micronutrient availability. We did not measure different ways of preparing cooked foods that might affect nutrient content (boiling vs. steaming) nor did we separate the consumption of cooked vs. canned foods. Further, factors such as soil deficiencies, fat consumption, storage methods, and the quality of produce can all influence the availability and absorption of micronutrients within the body. While these factors were beyond the scope of the current study, it is important to keep in mind that the way in which nutrients journey from the food to the brain is influenced by a multitude of factors, beyond raw vs. processed.

While the mood-conferring benefits of raw FV provide sufficient rationale for interventions, it should also be considered that the levels of poor mental health within this young adult sample require urgent attention. Young adults are considered at high risk of having poor mental health (Johnston et al., 2014), and the current sample was no exception. The mean depression score for the sample (*M* = 17.53) was above the 16-point clinical cut-off point on the CES-D, suggesting that young adults typically experience some symptoms of depression. The fact that this clinically significant level of depression was seen in the American MTurk participants (*M* = 18.07) and nearly seen for the New Zealand Psychology participants (*M* = 15.90) suggests that this phenomenon of young adult mental ill-health is pervasive, and does not necessarily reflect geographical or cultural environments. Given that young adults have high vulnerability to suffering from mental illness, and they also have the lowest fruit and vegetable consumption, the current results reaffirm young adults as an important population to target for mental health interventions, including those designed to improve diet.

## Conclusions

The current findings showed that consumption of raw fruits and vegetables differentially predicted better mental health than the consumption of processed fruits and vegetables even when controlling for demographic, socioeconomic, and health covariates. The cooking and processing of FV has the potential to diminish nutrient levels, which likely limits the delivery of nutrients that are essential for optimal emotional functioning. In term of application, our results suggest that policies, promotions, and interventions that are designed to increase raw fruit and vegetable consumption may provide an accessible adjuvant approach to improving mental health in the young adult population, who remain vulnerable to developing mental disorders.

## Author contributions

KB: Conceived the idea and co-wrote the manuscript; GB: Collected data and co-wrote the manuscript; TC: Conceived the idea, co-wrote the manuscript, and provided supervisory support to KB and GB. All authors gave their approval for the final manuscript.

### Conflict of interest statement

The authors declare that the research was conducted in the absence of any commercial or financial relationships that could be construed as a potential conflict of interest.

## Abbreviations

BMI: body mass index
FVI: fruit and vegetable intake
SES: socioeconomic status.
